# Characteristics and Clinical Value of 18F-FDG PET/CT in the Management of Adult-Onset Still’s Disease: 35 Cases

**DOI:** 10.3390/jcm10112489

**Published:** 2021-06-04

**Authors:** Josselin Brisset, Yvan Jamilloux, Stephanie Dumonteil, Guillaume Lades, Martin Killian, Mathieu Gerfaud-Valentin, Anne Lemaire, Tomasz Chroboczek, Eric Liozon, Guillaume Gondran, Pascal Sève, Jacques Monteil, Anne-Laure Fauchais, Kim Heang Ly

**Affiliations:** 1 Service de Médecine Interne, CHU Dupuytren, 87000 Limoges, France; Stephanie.Dumonteil@chu-limoges.fr (S.D.); eric.liozon@chu-limoges.fr (E.L.); Guillaume.GONDRAN@chu-limoges.fr (G.G.); anne-laure.fauchais@chu-limoges.fr (A.-L.F.); kim.ly@chu-limoges.fr (K.H.L.); 2 Service de Maladies Infectieuses et Tropicales, CHU Dupuytren, 87000 Limoges, France; 3 Service de Médecine Interne, Hôpital de la Croix-Rousse, 69004 Lyon, France; yvan.jamilloux@chu-lyon.fr (Y.J.); mathieu.gerfaud-valentin@chu-lyon.fr (M.G.-V.); pascal.seve@chu-lyon.fr (P.S.); 4 Service de Médecine Nucléaire, CHU Dupuytren, 87000 Limoges, France; guillaume.lades@chu-limoges.fr (G.L.); jacques.monteil@chu-limoges.fr (J.M.); 5 Service de Médecine Interne, CHU Saint-Etienne, 42055 Saint-Etienne, France; Martin.Killian@chu-st-etienne.fr; 6 Service de Médecine Interne, Centre Hospitalier Alpes-Léman, 74130 Contamine-sur-Arve, France; alemaire@ch-alpes-leman.fr; 7 Service de Maladies Infectieuses, Centre Hospitalier Alpes-Léman, 74130 Contamine-sur-Arve, France; tchroboczek@ch-alpes-leman.fr

**Keywords:** adult-onset Still’s disease, 18F-FDG PET/CT

## Abstract

While the diagnosis of adult-onset Still’s disease (AOSD) involves the exclusion of differential diagnoses, the characteristics and value of 18F-Fluorodeoxyglucose (18F-FDG) Positron Emission Tomography coupled with CT (PET/CT) in the management of AOSD remain poorly known. Our retrospective study included patients from four centers, fulfilling Yamaguchi or Fautrel criteria, who underwent a PET/CT during an active AOSD. Thirty-five patients were included. At the time of PET/CT, the Yamaguchi criteria were met in 23 of 29 evaluable cases. PET/CT showed bone marrow (74.3%), lymph node (74.3%), and splenic (48.6%) FDG uptake. Despite arthralgia or arthritis in most patients, joints were rarely the sites of 18F-FDG accumulation. The spatial distribution of 18F-FDG uptake was nonspecific, and its intensity could be similar to malignant disease. Lymph node or bone marrow biopsy was performed after PET/CT in 20 patients (57.1%). The intensity of bone marrow; splenic and lymph node hypermetabolism appeared to be correlated with disease activity. Abnormal PET/CT in the cervical lymph nodes and age ≥ 60 years seemed to be predictive factors for monocyclic evolution. The clinical value of PET/CT is not in direct diagnosis; but as an aid in excluding differential diagnoses by searching for their scintigraphic features and guiding biopsy.

## 1. Introduction

Adult-onset Still’s disease (AOSD) is a rare systemic multigenic autoinflammatory disorder, the pathogenesis of which remains poorly understood [1,2,3]. It is characterized by high spiking fever with transient skin rash, arthralgia or arthritis, sore throat, lymphadenopathy, hepatosplenomegaly, neutrophilic leukocytosis, hyperferritinemia with low glycosylated ferritin (<20%), and abnormal liver function test results [4]. AOSD is highly heterogeneous in its clinical presentation, severity, and outcomes [4,5]. The clinical course of the disease may have one of the following three patterns: a monocyclic systemic course, an intermittent or polycyclic systemic course, or a chronic articular course [3,4,5,6].

Due to the low prevalence of this pathology and the absence of specific clinical, radiological, and biological signs, the diagnosis of AOSD is often difficult and time-consuming. AOSD diagnosis is confirmed only after the exclusion of differential diagnoses, including infectious, inflammatory, and malignant diseases [7]. Excluding these differential diagnoses remains one of the most difficult challenges [8].

Although chest, abdominal, and pelvic computed tomography (CT) is almost always performed in the initial assessment of AOSD, the utility of 18F-fluorodeoxyglucose positron emission tomography coupled with CT (18F-FDG PET/CT) remains to be determined [7]. In cases of suspected AOSD—often in the context of fever of unknown origin (FUO) —18F FDG PET/CT is increasingly used by clinicians. However, there are few reports of the characteristics and usefulness of 18F-FDG PET/CT in AOSD, and only four reports have included more than 20 patients [9,10,11,12]. Moreover, the most recent studies were conducted mainly without the involvement of clinical physicians.

Therefore, the present study was performed to describe the characteristics of 18F-FDG PET/CT in AOSD patients during a flare, to identify the contribution of this examination to differential diagnosis, and to identify possible predictive factors of disease outcomes.

## 2. Patients and Methods

### 2.1. Patients

This multicenter retrospective study was performed in patients from four French centers: University Hospital Center of Limoges (Limoges, France), University Hospital of Lyon (Hospices Civils de Lyon, Lyon, France), University Hospital of Saint-Etienne (Saint-Priest-en-Jarez, France), and Alpes Léman Hospital Center (Contamine-sur-Arve, France).

Patients diagnosed with AOSD between 2005 and 2019 were identified by reviewing the database of the Medical Information Department. Patients were included if they fulfilled the Yamaguchi [13] or Fautrel [8] criteria and had undergone 18F-FDG PET/CT at the initial diagnostic assessment or during a disease flare. The exclusion criteria were insufficient medical record data and disease onset before 16 years old.

### 2.2. Data

The following data were collected using a standardized form: (1) clinical features: all manifestations observed in the flare during which 18F-FDG PET/CT was performed; (2) imaging features with a detailed description of 18F-FDG PET/CT results; (3) laboratory parameters measured at the time of disease flare, as close as possible to the time of 18F-FDG PET/CT when several data were available; (4) clinical course and treatments.

Laboratory tests, including routine blood tests, C-reactive protein (CRP), serum ferritin, rheumatoid factor, and anti-nuclear antibodies, were performed in all patients. To exclude malignant disease, some patients also underwent bone marrow or lymph node biopsy; their histological results were recorded.

Each patient was assessed for the systemic score proposed by Pouchot et al. for AOSD, with an assignment of 1 point to each of the following manifestations: fever, typical rash, pleuritis, pneumonia, pericarditis, hepatomegaly or abnormal liver function tests, splenomegaly, lymphadenopathy, leukocytosis > 15,000/mm^3^, sore throat, myalgia, and abdominal pain (range: 0–12) [14]. A modified systemic score with the addition of ferritin > 4000 ng/mL was also determined [15].

Three clinical courses were considered: monocyclic, defined as a single episode that faded and was followed by sustained clinical remission after 1 year or more of follow-up; polycyclic, defined as complete remission followed by one or more exacerbations; and chronic, defined as a persistently active disease, generally with polyarthralgia [6,14,16].

Data regarding the different treatment modalities were collected, with the associated clinical responses.

Clinical remission was defined as clinically asymptomatic AOSD with no biological inflammatory syndrome [6]. Corticosteroid-dependence was defined as AOSD recurrence despite a maintenance dose > 15 mg/day [6,16]. Corticosteroid resistance was defined as a clinical non-response to steroids, requiring the introduction of another agent.

### 2.3. 18F-FDG PET/CT Imaging and Interpretation

Different PET/CT instruments were used in the four centers participating in the study. However, all centers followed the same imaging process: before 18F-FDG injection, the patients fasted for at least 4 h and the blood glucose level was checked to ensure it was < 180 mg/dL; scans were performed 1 h after 18F-FDG injection (mean injection time: 72.1 ± 14.0 min). Whole-body CT and PET covered a region from the base of the skull to the mid-thigh. PET data were reconstructed by iterative methods and fused into PET/CT slices for evaluation. PET/CT was evaluated by a nuclear medicine physician according to the standard practices of each center.

18F-FDG uptake at characteristics sites (cervical, axillary, mediastinal, abdominopelvic, and inguinal lymph nodes, and bone marrow and spleen) and all other hypermetabolic sites were reported with two data: (1) PET result: a dichotomous variable indicating whether the 18F-FDG uptake was considered normal or abnormal by the nuclear medicine physician; (2) maximum standardized uptake value (SUVmax): calculated as the maximum activity concentration at the site based on injected dose and body weight. In cases with multiple lymphadenopathies at a given site, the highest SUVmax was selected. Ratios of SUVmax in the relevant sites to SUVmax in the liver were also used to standardize FDG uptake interpretation [11,12].

### 2.4. Statistical Analysis

All analyses were conducted using R software (version 3.2.2; R Foundation for Statistical Computing, Vienna, Austria).

For descriptive analysis, quantitative variables are presented as the means ± standard deviation (SD), and qualitative variables are shown as percentages. Descriptions were made considering the three groups, i.e., monocyclic, polycyclic, and chronic.

Comparisons between groups for qualitative variables were conducted using Pearson’s χ2 test if the application conditions were met; otherwise, a Fisher’s exact test was performed.

Intergroup comparisons of quantitative variables were conducted using Student’s *t*-test for variables following a normal distribution and the Wilcoxon or Kruskal–Wallis test for variables without a normal distribution.

The association between 18F-FDG uptake and biological data was evaluated using a Spearman correlation test.

To search for predictive factors for monocyclic evolution with logistic regression analysis, chronic course and polycyclic course were combined into a single group. Univariate analyses were performed between these two new groups. Variables with a *p* < 0.25 were included in a multivariate logistic model. Quantitative variables that met the assumption of linearity of the logit values were integrated without modification; otherwise, they were converted into qualitative variables. The initial multivariate model was simplified by a stepwise backward elimination method so that the final model included only variables significantly associated with the monocyclic evolution variable. The robustness of the model was evaluated using Pearson’s residuals and deviance residuals.

In all analyses, *p* < 0.05 was taken to indicate statistical significance.

### 2.5. Ethics Board Approval

All patient data were collected retrospectively. This study was conducted in compliance with Good Clinical Practices and the principles of the Declaration of Helsinki. In accordance with French law, formal approval from an ethics committee was not required for this type of retrospective study.

## 3. Results

### 3.1. Clinical and Laboratory Features

A total of 35 patients (20 women and 15 men) with a median age of 44 years (range: 17–72) were identified and included in the present study. Of these, 33 patients (94.3%) were admitted to hospital with FUO, and two patients (5.7%) were admitted for a flare of previously known AOSD. In the latter two patients, the reason for requesting PET/CT was doubt regarding the diagnosis after a flare with systemic signs.

The patients were all followed up at an internal medicine department or rheumatology department.

Table 1 shows patient characteristics and laboratory data.

The clinical course was monocyclic in 13 patients, polycyclic in 11 patients, and chronic in 8 patients. Three patients could not be categorized due to a lack of evolution data or insufficient follow-up at the time of inclusion. The patients with a polycyclic course were younger than the others, with a mean age of 34.0 ± 11.9 years (*p* = 0.031).

The main clinical manifestations were fever (100%), joint involvement (arthralgia or arthritis: 91.4%), skin rash (85.7%), odynophagia or pharyngitis (75%), and myalgia (62.9%).

Biological data showed hyperleukocytosis (77.1%), elevated serum ferritin (mean: 8635.6 ng/mL, range: 98–92,200), and glycosylated ferritin fraction < 20% in 21 out of 27 cases tested (78%).

There were no statistically significant clinical or biological differences between the three groups, except for hepatomegaly, which was more frequent in the polycyclic group (*p* = 0.004).

### 3.2. 18F-FDG PET/CT Characteristics

As presented in Table 2, increased 18F-FDG uptake was noted in 33 patients (94.3%). PET/CT showed homogeneous and intense accumulation of 18F-FDG in the bone marrow (74.3%) and spleen (48.6%).

Most patients (74.3%) had lymph node 18F-FDG accumulation. This accumulation involved the supradiaphragmatic lymph nodes (71.4%) more often than the infradiaphragmatic lymph nodes (48.6%). For 16 patients (45.7%), 18F-FDG accumulation was seen in the lymph nodes on both sides of the diaphragm. In six patients (17.1%), a left/right asymmetric distribution of lymphadenopathy was noted by the nuclear physician.

Only three patients had 18F-FDG accumulation in the joints; the involved joints were the shoulders, hips, and sternoclavicular joints.

Two patients had a normal 18F-FDG PET/CT: they had a biological inflammatory syndrome (CRP: 48 and 173 mg/l) but few clinical manifestations (systemic scores: 3 and 5).

**Figure 1:**
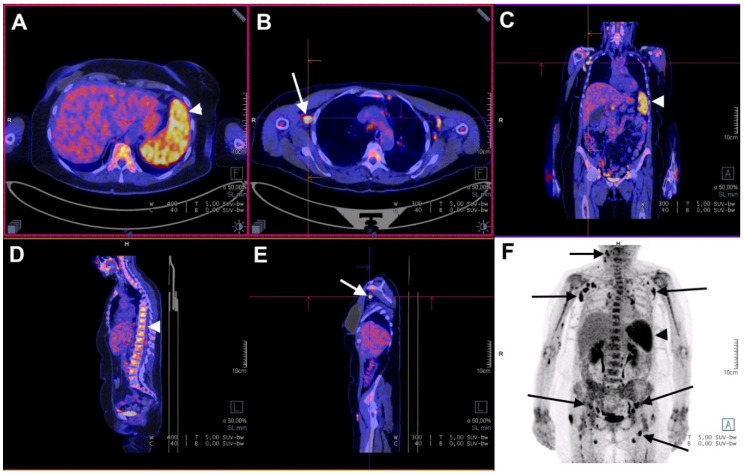
**18F-FDG PET/CT in a case of AOSD with a monocyclic course.** A 63-year-old woman presented with a fever up to 39.6 °C, asthenia, polyarthralgia, sore throat, and skin rash. Arrowheads show ^18^F-FDG accumulation in the spleen (**A**,**C**,**F**: SUV_max_ = 7.0) and in the bone marrow (**D**: SUV_max_ = 5.4). Arrows show 18F-FDG accumulation in multiple lymph nodes (**F**: SUV_max_ = 12.0). Moderate 18F-FDG accumulation is visible in the elbows and in the wrists. The lymph node with the highest SUV_max_ (**B**,**E**) was biopsied, and histological examination showed reactive hyperplasia without any signs of malignancy. The patient improved after aspirin treatment. Relapse was also treated with aspirin. At three months, there was no sign of disease activity, and no lymphadenopathy was seen on whole-body CT.

An example of 18F-FDG PET/CT in an AOSD patient is presented in Figure 1.

### 3.3. Correlation of 18F-FDG Uptake with Disease Activity

Abnormal PET/CT results in the lymph nodes were significantly correlated with a higher systemic score, higher ferritin level, and higher modified systemic score (Table 3). Abnormal bone marrow results were correlated with higher ferritin and CRP levels.

When searching for the correlations between quantitative PET measures and disease activity markers, spleen SUVmax was significantly correlated with the modified systemic score and ferritin (Table 4. When comparing SUVmax divided by liver SUVmax—with the liver used as a reference—almost all correlation coefficients increased, the bone marrow 18F-FDG uptake was correlated with ferritin and CRP values, and lymph node 18F-FDG uptake was correlated to the modified systemic score.

### 3.4. Clinical Management and Guidance for Biopsy

When 18F-FDG PET/CT was performed in the context of FUO evaluation, chest, abdominal, and pelvic CT scans had already been performed in 28 of the 33 patients. The Yamaguchi criteria were met in 23 of the 29 evaluable cases at this time.

As shown in Table 5, 18F-FDG PET/CT was followed by a lymph node biopsy in 11 patients. Among them, four showed no lymphadenopathy on whole-body CT. Bone marrow biopsies were performed in 13 patients.

For seven patients, the nuclear medicine physician suggested the possibility of lymphoma. Bone marrow biopsy was performed in three patients, and lymph node biopsy was performed in five patients (bone marrow or lymph node biopsy: five patients).

Histopathological examination showed no evidence of malignant pathology in any of the patients who underwent lymph node and bone marrow biopsy. All lymph nodes were described as showing reactive lymphadenitis, mostly with follicular lymphoid hyperplasia (Figure S1 in Supplementary Materials)

The bone marrow was described as rich, inflammatory, with a reactive appearance. One case, with massive 18F-FDG accumulation in bone marrow, spleen and lymph nodes, showed hemophagocytosis on bone marrow biopsy.

### 3.5. Treatment, Response to Treatment, and Outcome

Seven patients were treated with nonsteroidal anti-inflammatory drugs only. Data were missing for three patients.

Corticosteroid treatment was required in 25 patients. Among these patients, corticosteroid dependence was observed in 1/9 in the monocyclic group, 4/9 in the polycyclic group, and 7/7 in the chronic group; as expected, the difference between these evolution courses was significant (*p* = 0.002). Corticosteroid resistance was observed in 1/9 patients, 2/9 patients, and 2/7 patients in the monocyclic, polycyclic, and chronic groups, respectively.

Biotherapy targeting interleukin-1 (IL-1) was used in 10 patients (two in the monocyclic group, five in the polycyclic group, and three in the chronic group), always in combination with corticosteroids, with complete remission of the flare in seven cases.

With the exception of corticosteroid dependence, there were no significant differences in the above-mentioned treatments between the three groups.

In the univariate analyses, 18F-FDG PET/CT data were not significantly associated with evolution course, corticosteroid dependence, or corticosteroid resistance.

### 3.6. Predictive Factors of Evolution

Logistic regression analyses were performed to determine factors associated with monocyclic evolution compared with non-monocyclic evolution (polycyclic and chronic courses).

At equivalent age, cervical lymph node hypermetabolism in 18F-FDG PET/CT was significantly associated with monocyclic evolution (odds ratio: 0.92, 95% confidence interval: 0.8–1.0, *p* = 0.024).

By selecting an age cut-off of 60 years, the logistic regression model showed that patients with age ≥ 60 years and abnormal cervical lymph node 18F-FDG PET results were most likely to have monocyclic evolution of their disease (Table 6). Age < 60 years and no abnormality in the cervical lymph node 18F-FDG PET was therefore significantly associated with a higher risk of non-monocyclic evolution.

## 4. Discussion

The present study was performed to analyze the characteristics of PET/CT during a flare of AOSD, its correlations with disease activity, its contribution to the exclusion of differential diagnoses, and to identify predictive factors of disease outcomes.

This study of 35 patients undergoing PET/CT during active AOSD describes for the first time the entire care process from diagnosis to response to treatment.

The clinical and biological characteristics of our patients were similar to those in the main series on AOSD [6,8,12,14,17,18,19,20,21,22]. Slight female predominance, constant fever, arthromyalgia, skin rash, and sore throat were found in most patients. Whereas most studies reported age at diagnosis below 40 years, in this study, the age appeared to be higher, with a mean of 46 years (median: 44 years). This may have been because PET/CT, in an FUO context, was more commonly justified by the higher risk of malignant disease in older patients. However, the cohort of AOSD patients described by Ruscitti et al. [22]—a recent study—had a very similar age at diagnosis (mean: 45 years) without any selection on the tests performed.

PET/CT showed homogeneous and diffuse bone marrow hypermetabolism in 75% of the patients, confirming data from the main published studies, in which the bone marrow intensely fixed the marker in 80%–100% of cases [9,10,11,12,23,24,25]. This diffuse uptake is probably related to inflammatory parameters reflecting the cellular activation within the bone marrow [26]. This may be due to the increased frequency of occult macrophage activation syndrome during active AOSD [12,27,28,29]. Actually, one of our cases had hemophagocytosis on bone marrow biopsy.

The examinations showed at least one hypermetabolic lymph node site at the same rate as in the published data (67–84% of cases) [9,11,12,24,25]. Hypermetabolic lymph nodes were preferentially supradiaphragmatic in our study. This characteristic had already been noted by Jiang et al. [10]. Lymph node hypermetabolism generally appeared symmetrical (83% of patients), but exceptions were possible. In one of our cases, a clearly asymmetrical characteristic was noted by the nuclear medicine physician. AOSD was diagnosed after obtaining the results of lymph node biopsy and exclusion of malignant disease, followed by a favorable outcome with corticosteroid therapy.

Homogeneous and diffuse accumulation of 18F-FDG in the spleen was the third most common characteristic; as in the study of An et al. [24], it was observed in half of our patients, while other studies reported this result in more than 80% of cases [10,11,24].

Notably, while arthralgia or arthritis was reported in more than 90% of patients, 18F-FDG accumulation in the joints was an exception in our study. This finding was consistent with the findings of Jiang et al. [10], in which only 3% of patients showed joint hypermetabolism and those of Zhou et al. (9% versus 24% for other connective tissue diseases) [11]. These results suggest that despite the clinical involvement, major joint 18F-FDG uptake is not characteristic of AOSD PET/CT. However, Dong et al. [9] reported a much higher rate of joint 18F-FDG uptake (54%), which could be explained by the choice of a low cut-off value to consider SUVmax as pathological (SUVmax between 1.20 and 3.15). These data contrast with what is known in rheumatoid arthritis, where joint FDG uptake reflects the disease activity, with strong correlations between FDG uptake and clinical parameters [30,31,32,33].

Although sore throat was a common symptom, it was associated with pharyngeal hypermetabolism in only one case. This could be explained by the fact that sore throat is often temporary in AOSD.

These descriptive data did not reveal a specific profile of AOSD compared to other diseases with similar PET/CT, such as lymphoma [10,11,24].

As reported by Yamashita et al., the 18F-FDG uptake observed in AOSD ranged in intensity that may be compatible with both benign and malignant disease [23].

In our study, SUVmax reached 7.0 in the spleen, 9.0 in the bone marrow, and 18.9 in the lymph nodes, which were comparable to those of malignant diseases, such as lymphoma [26].

Yamashita et al. compared their data with PET/CT of inflammatory rheumatism diseases; FDG accumulation levels in bone marrow and spleen were higher in AOSD than in rheumatoid arthritis and spondyloarthropathy [23]. Zhou et al. showed that AOSD patients had higher uptake of FDG in the spleen, bone marrow, and lymph nodes than patients with other connective tissue diseases [11].

In a study of 13 patients, An et al. [24] showed a good correlation between bone marrow, splenic, and lymph node 18F-FDG uptake and clinical disease activity assessed by the systemic score of Pouchot et al. [14]. A recent study showed that this score could predict disease severity [22], and it has since been improved by Rau et al. [34] by the addition of ferritin level, a known biomarker of disease activity [15]. The same study revealed correlations with biomarkers of inflammation. Conversely, Dong et al. did not detect correlations with biological data [9].

Our study showed correlations between SUVmax of the spleen, bone marrow, and lymph nodes and the modified systemic score. Correlations appeared to be strengthened when values were reported for liver SUV, probably by excluding technical bias. Our study probably did not have sufficient power to show a stronger correlation. Wan et al. [12] also reported correlations between hypermetabolism of the spleen, bone marrow, lymph nodes, and the systemic score.

These correlations suggest that PET/CT may have a place in the monitoring of AOSD, even though the disease is usually monitored by clinical and biological surveillance [35]. Yamashita et al. [23] and Choe et al. [36] showed that clinical improvement was associated with a decrease in 18F-FDG uptake in all cases tested.

As outlined above, the spatial distribution of 18F-FDG uptake did not show specific characteristics. SUVmax of patients with AOSD could reach levels of malignant diseases, and PET/CT results could mimic those of lymphoma.

In a recent prospective study on the value of PET/CT in identifying the causes of FUO or inflammation of unknown origin [37], AOSD (as defined by Yamaguchi criteria plus biological parameters) appeared to be the first cause of FUO (15.3%). In the same study, PET/CT was classified as non-helpful for diagnosis of AOSD, while imaging analysis showed 18F-FDG accumulation in the bone marrow and lymph nodes due to their nonspecific characteristics.

Therefore, while PET/CT has proven diagnostic value in other inflammatory diseases by highlighting specific disorders (aortitis in giant cell arteritis [38], paraaortic tissue hypermetabolism in IgG4-related disease [39]), it was not helpful for a positive diagnosis of AOSD.

Lymph node or bone marrow biopsies were performed after the PET/CT in most cases in our study. For the 11 lymph node biopsies (31.4% of patients), clinicians used the PET/CT to choose the most hypermetabolic site among those that could be reached. Biopsied lymph nodes had higher SUVmax than those of non-biopsied patients (9.7 ± 4.5 vs. 6.0 ± 2.1, respectively; *p* = 0.03). This can be interpreted either as a factor influencing the biopsy decision or as an element of a more suggestive picture of hematological disease.

Among the lymph node biopsies, four (36%) were at sites not suggested to show lymphadenopathy on whole-body CT but were chosen because they showed the highest level of 18F-FDG accumulation.

The histology of biopsy specimens allowed exclusion of malignant diseases and finalization of the diagnosis of AOSD. It usually indicated the presence of reactive inflammatory bone marrow and/or reactive lymph node hyperplasia (Figure S1 in Supplementary Data).

These data raise several questions, including whether more biopsies are performed after PET/CT; whether these biopsies, which improve the diagnosis by excluding hematological diseases, are reasonable; and whether more diagnostic errors occur when PET is not used. Further large-scale prospective studies are required to address these questions.

PET/CT could sometimes avoid unnecessary biopsy [9,10]. In this way, two of our patients, despite hypermetabolic lymphadenopathies above and below the diaphragm, were not biopsied. The clinical and biological data were very suggestive of AOSD, and FDG uptake was almost symmetrical, leading the clinician to this logical decision.

Our study indicated that even though Yamaguchi’s criteria [13] were met before the PET/CT request in the vast majority of evaluable cases (i.e., available laboratory data and whole-body CT performed), the clinician did not consider them sufficient to establish the diagnosis. This is a defining feature of AOSD, a positive diagnosis of which is based on the exclusion of differential diagnoses. However, this exclusion is never easy. Furthermore, there are also forms of paraneoplastic pseudo-AOSD. Liozon et al. reported a case with a clinical presentation very suggestive of AOSD, in which, despite an initial response to corticosteroid, the AOSD-like syndrome appeared to be a metastatic melanoma [40].

Difficulty in making the diagnosis of AOSD was also shown in the diagnostic delay, which was about 4.5 months in our study, as reported in a recent series of patients with AOSD [41]. To reduce the delay to diagnosis with PET/CT, it would be necessary to have faster access to this examination; in our study, PET/CT was prescribed mainly as a second line option.

Before diagnosis, the clinical presentation was FUO. Several studies have shown that PET/CT improves the diagnosis rate of FUO [37,42,43]. A recent study by Schönau et al. showed that age > 50 years and CRP level > 30 mg/L were predictive factors for the diagnostic utility of PET/CT, suggesting that these groups are most likely to benefit from this examination [37]. Therefore, in a situation where AOSD is only one hypothesis among a number of others, PET/CT is a valuable FUO diagnostic strategy. It could be associated with a significant reduction in the diagnostic delay, especially when performed early, in selected patients [37].

Taken together, the findings outlined above suggest that, when AOSD becomes a credible diagnostic hypothesis, the role of PET/CT can be summarized as follows:

− It does not provide direct utility for a positive diagnosis, given its nonspecific characteristics;
− Its diagnostic usefulness is in the exclusion of differential diagnoses; by making the diagnostic hypothesis of a solid tumor highly improbable, and combined with clinical and biological data, it often reduces the likely diagnostic hypothesis to AOSD or lymphoma;
− In the latter case, if there is any doubt, a biopsy could be performed to allow the exclusion of hematological disease, and PET/CT may be a help to choose the biopsy site.

Our study showed that abnormal PET in the cervical lymph nodes and age > 60 years were predictive factors for monocyclic evolution. Conversely, age < 60 years and normal cervical PET appeared to be associated with a more complicated, recurrent (polycyclic) or progressing to a chronic clinical course.

The cervical lymph nodes were also the sites showing the most intense tracer accumulation.

The retrospective nature of our study resulted in missing data. These concerned follow-up in a few cases, and more often PET/CT data; quantitative measurements of hypermetabolism were not always available, particularly for bone marrow, where SUVmax was not reported for most cases because of its lack of utility in clinical practice.

The measurement of liver SUVmax as a reference for reporting other SUVmax values was not available for all patients. However, SUV is influenced by different factors, including scanner, scatter and attenuation correction, and the reconstruction algorithm used. Standardizing the SUV to a reference area, such as the liver, would avoid most technical biases [44] and facilitate comparisons among centers.

There may have been selection bias by conditional inclusion in PET/CT, although the clinical and biological characteristics of the patients appeared comparable to those of AOSD studies.

Further large-scale prospective studies comparing patients with and without early PET/CT are needed to reach conclusions about the utility of PET/CT to reduce the diagnostic delay and the number of investigations required to exclude differential diagnoses.

Hodgkin’s and non-Hodgkin’s lymphomas appear to be the main differential diagnoses of AOSD, and concerns regarding not excusing these diseases lead to many bone marrow and lymph node biopsies. Therefore, it would seem relevant to compare our study with a lymphoma PET/CT series to highlight the differences, if any, or their absence.

With targeted biotherapies with spectacular efficacy in AOSD [7,45,46], when a diagnosis of lymphoma is not clearly excluded, PET/CT may be useful to evaluate response to treatment. Therefore, comparative PET/CT may be useful when no biopsy has been performed to confirm a diagnosis of AOSD by evaluating the response to anti-IL-1 biotherapy in addition to the clinical and biological assessment.

Finally, in every relapse, a reassessment in search of an infectious or malignant trigger is usual: PET could find a rightful place in this situation.

## 5. Conclusions

PET/CT was almost always abnormal in patients with active AOSD (94.3%), with bone marrow, splenic, and/or lymph node hypermetabolism. The spatial distribution of 18F-FDG uptake was nonspecific, and its level could mimic a malignant disease. The intensity of FDG uptake in the bone marrow, splenic, and lymph nodes appeared to be correlated with disease activity, suggesting its usefulness in disease and treatment response monitoring.

The clinical value of PET/CT in AOSD is not in direct diagnosis but as an aid in excluding differential diagnoses by searching for their scintigraphic features. Lymph node or bone marrow biopsies were performed after the PET/CT in most of our patients. PET/CT may be valuable for predicting the disease outcome. Further studies are needed to confirm these results.

## Figures and Tables

**Table 1:** Patient characteristics and laboratory data.

	All Patients *n* = 35		Monocyclic *n* = 13/32 ^¥^		Polycyclic *n* = 11/32 ^¥^		Chronic *n* = 8/32 ^¥^		*p*-Value
	Number (%) or Mean ± SD								
Female	20	(57.1)	10	(76.9)	6	(54.5)	4	(50.0)	NS
Male	15	(42.9)	3	(23.1)	5	(45.5)	4	(50.0)	NS
Age (year), mean ± SD	46.2 ± 16.5		52.5	±16.1	34.0	±11.9	50.1	±17.3	0.031
Delay to diagnostic (months); mean (range)	4.5	(1–34)	4.5	(1–34)	3	(1–14)	7.5	(1–18)	0.035
*Clinical features*									
Fever	35	(100)	13	(100)	11	(100)	8	(100)	NS
Fever ≥ 39 °C *	28/32	(87.5)	11	(84.6)	9	(81.8)	7	(87.5)	NS
Arthralgia/arthritis	32	(91.4)	12	(92.3)	11	(100.0)	8	(100.0)	NS
Rash	30	(85.7)	11	(84.6)	9	(81.8)	7	(87.5)	NS
Sore throat/pharyngitis *	24/32	(75.0)	12	(92.3)	7	(63.6)	5	(62.5)	NS
Myalgia	22	(62.9)	6	(46.2)	7	(63.6)	6	(75.0)	NS
Clinical lymphadenopathy	12	(34.3)	5	(38.5)	4	(36.4)	3	(37.5)	NS
Clinical hepatomegaly	6	(17.1)	0	(0)	5	(45.5)	0	(0)	0.004
Clinical splenomegaly *	5	(15.6)	1	(7.7)	4	(36.4)	0	(0)	NS
*Laboratory findings*									
White blood cells > 10 G/L *	27	(77.1)	9	(69.2)	10	(90.9)	7	(87.5)	NS
Polymorphonuclear cells ≥ 80%	19/28	(67.9)	6/11	(54.5)	9/10	(90)	4/7	(57.1)	NS
CRP (mg/L); mean (range)	137.8	(17.4–417)	131.3	(25–373)	155.4	(17.4–417)	129.1	(20–391)	NS
Ferritin (ng/mL); mean (range)	8635	(98–92,200)	14,710	(98–92,200)	5859	(108–22,800)	1507	(107–2800)	NS
Glycosylated ferritin ≤ 20% *	21/27	(78)	9/11	(81.8)	7/8	(87.5)	5	(63)	NS
Elevated liver enzymes	15	(42.9)	6	(46.2)	6	(54.5)	2	(25.0)	NS
Negative for rheumatoid factor	35	(100)	13	(100)	11	(100)	8	(100)	NS
Antinuclear antibodies = 1/160 *	5/33	(15.2)	2	(16.7)	1/10	(10.0)	1	(12.5)	NS
Antinuclear antibodies > 1/160 *	0/33	(0)	0	(0)	0	(0)	0	(0)	NS
Systemic score, mean ± SD	5.2 ± 1.4	5.1 ± 1.4			5.6 ± 1.7	4.9 ± 0.8			NS

Number (percentage) unless otherwise stated; * Number positive/number tested (percentage); ¥ Number positive/number tested; three patients could not be classified due to a lack of evolution data; CRP: C-reactive protein; Systemic score (range: 1–12) proposed by Pouchot et al. [14] assigns one point for each of the following manifestations: fever, typical rash, pleuritis, pneumonia, pericarditis, hepatomegaly or abnormal liver function tests, splenomegaly, lymphadenopathy, leukocytosis > 15,000/mm^3^, sore throat, myalgia, and abdominal pain.

**Table 2:** 18F-FDG-PET/CT characteristics in 35 AOSD patients.

Abnormal PET/CT	33/35 (94.3%)			
Abnormal 18F-FDG Uptake Site	Number (%)	SUV Available	SUV_max_, Mean ± SD	SUV_max_, Range
**Bone marrow**	26/35 (74.3)	9	6.3 ± 1.9	3.9–9.0
**Spleen**	17/35 (48.6)	13	4.2 ± 1.5	2.6–7.0
**Lymph node**				
All sites	26/35 (74.3)	24	7.7 ± 3.9	3.3–18.9
Supradiaphragmatic	25/35 (71.4)	23	7.0 ± 3.7	2.7–16.3
Cervical	16/35 (45.7)	12	7.4 ± 3.3	2.8–13.5
Axillary	15/35 (42.9)	13	6.8 ± 4.5	1.6–16.3
Mediastinal	23/35 (65.7)	19	5.7 ± 2.2	2.6–10.7
Infradiaphragmatic	17/35 (48.6)	15	7.0 ± 4.4	2.8–18.9
Abdominal and pelvic	16/35 (45.7)	14	6.6 ± 4.0	2.8–18.9
Inguinal	7/35 (20.0)	4	6.7 ± 5.2	2.4–14.0
**Other areas**				
Joint	3/35 (8.6)	2	6.5 ± 4.7	3.1–9.8
Lung	4/35 (11.4)	1	4.5	
Muscle	2/35 (5.7)	1	3.0	

Reported in one case only: pleura, pericardium, thyroid cartilage, tonsils, stomach, and colon.

**Table 3:** 18F-FDG uptake results compared with clinical and biological disease activity.

18F-FDG Uptake ^¥^	*p*-Value				
	Systemic Score	Systemic Score + Ferritin > 4000	Ferritin	CRP	White Blood Cells
Spleen ^¥^	0.122	0.057	0.080	0.269	0.597
Bone marrow ^¥^	0.249	0.201	0.004 ^$^	0.040 ^$^	0.706
Lymph nodes ^¥^	0.007 ^$^	0.005 ^$^	0.021 ^$^	0.955	0.678

The data were assessed using Wilcoxon tests. ^$^ bold font: statistically significant. ^¥^ The result is a dichotomous variable indicating whether the area’s 18F-FDG uptake was considered normal or abnormal by the nuclear medicine physician.

**Table 4:** Correlation between 18F-FDG uptake and clinical and biological disease activity.

18F-FDG Uptake	Systemic Score		Systemic Score + Ferritin > 4000		Ferritin		CRP		White Blood Cells	
	*ρ*	*p*-Value	*ρ*	*p*-Value	*ρ*	*p*-Value	*ρ*	*p*-Value	*ρ*	*p*-Value
Spleen SUV_max_	0.440	0.128	0.566	**0.049^$^**	0.688	**0.017 ^$^**	0.236	0.413	0.001	0.996
Spleen/Liver ratio *	0.431	0.135	0.534	0.064	0.765	**0.003 ^$^**	0.532	**0.039 ^$^**	0.396	0.125
Bone marrow SUV_max_	0.287	0.416	0.425	0.229	0.575	0.104	0.467	0.187	−0.133	0.706
Bone marrow/Liver ratio *	0.584	0.065	0.616	0.052	0.553	**0.046 ^$^**	0.745	**0.007 ^$^**	0.324	0.243
Lymph nodes SUV_max_	0.017	0.939	0.109	0.618	0.101	0.629	−0.246	0.238	−0.109	0.600
Lymph nodes/Liver ratio *	0.446	0.108	0.593	**0.032 ^$^**	0.437	0.115	0.120	0.666	−0.077	0.782

The data were assessed using the Spearman correlation test. ^$^ bold font: statistically significant. * SUV_max_ divided by the liver SUV_max;_ *ρ*: Spearman’s rank correlation coefficient; 18F-FDG: 18F-Fluorodeoxyglucose; CRP: C-reactive protein; Systemic score (range: 1–12) proposed by Pouchot et al. [14] assigns one point for each of the following manifestations: fever, typical rash, pleuritis, pneumonia, pericarditis, hepatomegaly or abnormal liver function tests, splenomegaly, lymphadenopathy, leukocytosis > 15,000/mm^3^, sore throat, myalgia, and abdominal pain.

**Table 5:** Post 18F-FDG PET/CT biopsy.

	*n* = 35 *	SUV_max_^$^
Lymph node biopsy	11 (31.4)	9.7 ± 4.6
Lymph node biopsy with no lymphadenopathy on CT-TAP	4 (11.4)	7.4 ± 3.9
Bone marrow biopsy	13 (37.1)	5.4 ^¥^
Lymph node or bone marrow biopsy	20 (57.1)	n/a

* Number (percentage); ^$^ Mean ± standard deviation; **^¥^** Mean of two available values. CT-TAP: Computed tomography of the thorax, abdomen, and pelvis.

**Table 6:** Logistic regression analyses to determine factors associated with monocyclic evolution.

	β	OR	95% CI	*p*-Value
Age ≥ 60 years	2.9	19.0	2.6–405.1	0.0129
Abnormal cervical lymph nodes ^18^F-FDG PET result	2.5	11.6	1.7–237.0	0.0329

The model had a sensitivity of 84.2% and specificity of 61.5%; area under the curve = 0.83. OR: odds ratio; CI: confidence interval.

## Data Availability

The data presented in this study are available on reasonable request from the corresponding author.

## Supplementary Materials

The following are available online at https://www.mdpi.com/article/10.3390/jcm10112489/s1, Figure S1: 18F-FDG PET/CT-guided lymphadenopathy biopsy.
