# Supplementary material for: Characteristics and Clinical Value of 18F-FDG PET/CT in the Management of Adult-Onset Still’s Disease: 35 Cases
Source: J Clin Med. 2021 Jun 4;10(11):2489. doi: 10.3390/jcm10112489 (PMC8200084; doi:10.3390/jcm10112489)
Supplement: Supplementary file 1 [file jcm-10-02489-s001.zip › jcm-1205676-supplementary.pdf]

Supplementary Data

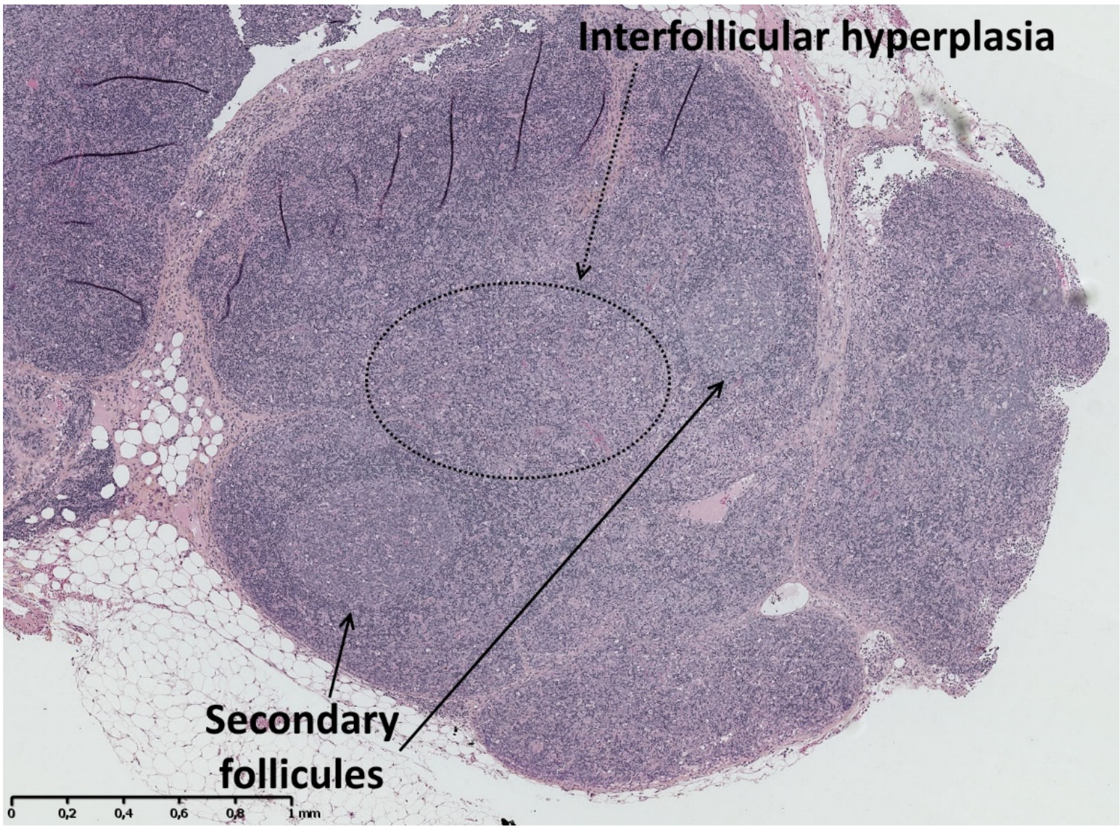

Figure S1. ^{18}F-FDG PET/CT-guided lymphadenopathy biopsy. Typical nonspecific histology found in AOSD mimicking lymphoma: paracortical hyperplasia with immunoblastic reaction. No suggestion of malignant pathology.
